# Supplementary material for: Scaffold Repurposing Reveals New Nanomolar Phosphodiesterase Type 5 (PDE5) Inhibitors Based on Pyridopyrazinone Scaffold: Investigation of In Vitro and In Silico Properties
Source: Pharmaceutics. 2022 Sep 15;14(9):1954. doi: 10.3390/pharmaceutics14091954 (PMC9501832; doi:10.3390/pharmaceutics14091954)
Supplement: Supplementary file 1 [file pharmaceutics-14-01954-s001.zip › pharmaceutics-1872916-supplementary.pdf]

Scaffold Repurposing Reveals New Nanomolar Phosphodiesterase Type 5 (PDE5) Inhibitors Based on Pyridopyrazinone Scaffold: Investigation of In Vitro and In Silico Properties

Kamelia M. Amin [1], Ossama M. El-Badry [2], Doaa E. Abdel Rahman [1], Magda H. Abdellattif [3], Mohammed A.S. Abourehab [4,5], Mahmoud H. El-Maghrabey [6], Fahmy G. Elsaid [7,8], Mohamed A. El Hamd [9,10,*], Ahmed Elkamhawy [11,12,*] and Usama M. Ammar [13,*]

[1] Pharmaceutical Chemistry Department, Faculty of Pharmacy, Cairo University, Cairo, 11562, Egypt
[2] Pharmaceutical Chemistry Department, Faculty of Pharmacy, Ahram Canadian University (ACU), Giza, 12566, Egypt
[3] Department of Chemistry, College of Science, Taif University, P.O. Box 11099, Taif 21944, Saudi Arabia
[4] Department of Pharmaceutics, College of Pharmacy, Umm Al-Qura University, Makkah 21955, Saudi Arabia
[5] Department of Pharmaceutics and Industrial Pharmacy, College of Pharmacy, Minia University, Minia 61519, Egypt
[6] Department of Pharmaceutical Analytical Chemistry, Faculty of Pharmacy, Mansoura University, 35516 Mansoura, Egypt
[7] Biology Department, Science College, King Khalid University, Abha, Saudi Arabia
[8] Zoology Department, Faculty of Science, Mansoura University, Mansoura, Egypt
[9] Department of Pharmaceutical Sciences, College of Pharmacy, Shaqra University, Shaqra 11961, Saudi Arabia
[10] Department of Pharmaceutical Analytical Chemistry, Faculty of Pharmacy, South Valley University, Qena 83523, Egypt
[11] BK21 FOUR Team and Integrated Research Institute for Drug Development, College of Pharmacy, Dongguk University-Seoul, Goyang 10326, Korea
[12] Department of Pharmaceutical Organic Chemistry, Faculty of Pharmacy, Mansoura University, Mansoura 35516, Egypt
[13] Strathclyde Institute of Pharmacy and Biomedical Sciences, University of Strathclyde, 161 Cathedral Street, Glasgow G4 0NR, Scotland, United Kingdom
[*] Correspondence: usama.ammar@strath.ac.uk (U.M.A.), a_elkamhawy@mans.edu.eg (A.E.) and aboelhamdmohamed@su.edu.sa (M.A.E.)

Supplementary Materials

Table of content

**S1.** Training set	Page 2
**S2.** Test set	Page 6
**S3.** Synthetic schemes	Page 9
**S4.** Chemical experimental	Page 10
**S5.** Molecular docking screening	Page 18
**S6.** Molecular dynamic (MD) simulation	Page 18
**S7.** References	Page 19

S1. Training set

150 reported PDE5 inhibitors (**t1–150**) were used as training set in this study

Table S1. Key structure of training set (**t1–150**)

**Training set**		**PDE5 IC_{50} (nM)**		**Ref.**
**Compound code**	**Compound code in Ref.**	**Experimental**	**Predicted**	
**t1 (1)**	Sildenafil	2.2	30.0	[1]
**t2 (2)**	Vardenafil	1.0	30.7	[1]
**t3 (3)**	Tadalafil	1.2	22.9	[1]
**t4 (4)**	18	13.4	21.9	[2]
**t5**	Benzamidenafil	1.1	24.8	[3]
**t6**	Avanafil	5.2	16.5	[5]
**t7**	Homosildenafil	3.8	31.3	[5]
**t8**	Hydroxyhomosildenafil	3.4	29.3	[5]
**t9**	Acetildenafil	7.6	15.7	[5]
**t10**	Hydroxyacetildenafil	58.4	100.7	[5]
**t11**	Thiosildenafil	0.6	13.5	[5]
**t12**	Carbodenafil	5.2	29.5	[6]
**t13**	Udenafil	8.3	24.7	[6]
**t14**	Norneosildenafil	5.8	16.6	[3]
**t15**	Thioaildenafil	0.6	45.5	[3]
**t16**	Nitrodenafil	67.4	61.8	[3]
**t17**	Dimethylacetildenafil	51.2	54.6	[3]
**t18**	Zaprinast	300.0	421.4	[7]
**t19**	9a	3.0	23.2	[4]
**t20**	9b	11.0	48.8	[4]
**t21**	10	3.0	21.2	[4]
**t22**	6c	150.0	253.0	[8]
**t23**	6e	60.0	88.7	[8]
**t24**	6f	70.0	93.8	[8]
**t25**	6h	100.0	272.9	[8]
**t26**	6i	140.0	101.0	[8]
**t27**	6l	50.0	95.5	[8]
**t28**	6m	370.0	126.5	[8]
**t29**	6n	320.0	384.4	[8]
**t30**	6o	70.0	88.8	[8]
**t31**	6q	530.0	145.6	[8]
**t32**	6r	200.0	163.2	[8]
**t33**	6s	510.0	358.6	[8]
**t34**	6t	500.0	141.6	[8]
**t35**	6u	38.0	38.3	[8]
**t36**	6v	310.0	408.4	[8]
**t37**	27	0.3	18.2	[2]
**t38**	28	0.3	16.3	[2]
**t39**	30	0.3	12.3	[2]
**t40**	6	0.3	13.3	[9]
**t41**	29	0.4	19.4	[2]
**t42**	26	0.9	15.7	[2]

**Training set**		**PDE5 IC_{50} (nM)**		**Ref.**
**Compound code**	**Compound code in Ref.**	**Experimental**	**Predicted**	
**t43**	25	1.0	20.7	[2]
**t44**	9	1.3	20.9	[2]
**t45**	17	1.6	28.9	[2]
**t46**	10	2.0	31.0	[2]
**t47**	35	2.3	20.0	[2]
**t48**	36	2.4	28.0	[2]
**t49**	11	2.5	30.1	[2]
**t50**	13	2.7	19.1	[2]
**t51**	31	2.9	47.1	[2]
**t52**	15	3.0	31.2	[2]
**t53**	19	3.5	27.3	[2]
**t54**	16	4.6	20.5	[2]
**t55**	34	14.5	60.9	[2]
**t56**	12	20.3	32.0	[2]
**t57**	22	22.4	20.1	[2]
**t58**	37	26.7	20.1	[2]
**t59**	14	28.5	40.2	[2]
**t60**	23	33.3	103.2	[2]
**t61**	33	33.9	36.2	[2]
**t62**	32	36.5	39.3	[2]
**t63**	20	52.5	90.6	[2]
**t64**	21	149.0	214.0	[2]
**t65**	24	224.0	375.2	[2]
**t66**	38	534.0	162.7	[2]
**t67**	2	0.9	18.7	[9]
**t68**	3	1.1	17.8	[9]
**t69**	4	10.6	23.8	[9]
**t70**	5	0.1	13.0	[9]
**t71**	1	51.0	57.5	[2]
**t72**	2	25.0	24.1	[2]
**t73**	3	43.0	94.4	[2]
**t74**	9	4.0	21.4	[10]
**t75**	10	0.8	15.6	[10]
**t76**	11	16.3	68.9	[10]
**t77**	12	0.3	14.3	[10]
**t78**	13	1.1	15.8	[10]
**t79**	14	1.2	21.9	[10]
**t80**	15	0.9	14.7	[10]
**t81**	16	0.2	30.2	[10]
**t82**	17	0.1	20.2	[10]
**t83**	18	0.1	15.1	[10]
**t84**	19	0.1	15.2	[10]

Training set		PDE5 IC_{50} (nM)		Ref.
**Compound code**	**Compound code in Ref.**	**Experimental**	**Predicted**	
**t85**	20	3.0	22.3	[10]
**t86**	21	1.1	30.8	[10]
**t87**	22	2.6	16.1	[10]
**t88**	23	0.7	12.6	[10]
**t89**	24	0.4	13.4	[10]
**t90**	25	0.1	17.1	[10]
**t91**	26	0.3	14.4	[10]
**t92**	27	0.1	19.2	[10]
**t93**	7a	0.3	23.4	[1]
**t94**	7b	0.4	15.5	[1]
**t95**	7c	4.3	18.4	[1]
**t96**	7d	15.0	30.9	[1]
**t97**	7e	4.1	61.4	[1]
**t98**	7f	0.6	12.6	[1]
**t99**	16f	410.0	257.5	[7]
**t100**	14h	160.0	158.1	[7]
**t101**	16h	65.0	102.8	[7]
**t102**	21d	350.0	146.5	[7]
**t103**	5a	9.3	23.8	[4]
**t104**	5b	4.9	21.5	[4]
**t105**	6	86.0	68.9	[4]
**t106**	7a	0.6	13.6	[4]
**t107**	7b	0.5	16.5	[4]
**t108**	11	17.0	53.0	[4]
**t109**	12	240.0	340.3	[4]
**t110**	13	190.0	354.2	[4]
**t111**	34a	177.0	123.1	[4]
**t112**	35c	235.0	247.3	[4]
**t113**	37a	52.5	100.6	[4]
**t114**	37b	39.7	61.4	[4]
**t115**	41	227.0	447.2	[4]
**t116**	Aminotadalafil	4.2	20.4	[5]
**t117**	4a	290.0	170.3	[11]
**t118**	4d	38.0	46.4	[11]
**t119**	7a	100.0	259.0	[11]
**t120**	7d	60.0	89.7	[11]
**t121**	8a	320.0	433.4	[11]
**t122**	8d	50.0	97.5	[11]
**t123**	9a	260.0	124.3	[11]
**t124**	9d	50.0	67.5	[11]
**t125**	10d	40.0	65.4	[11]
**t126**	8a	160.0	277.1	[4]

**Training set**		**PDE5 IC_{50} (nM)**		**Ref.**
**Compound code**	**Compound code in Ref.**	**Experimental**	**Predicted**	
**t127**	8b	170.0	169.1	[4]
**t128**	19b	36.9	25.3	[4]
**t129**	19c	12.7	24.9	[4]
**t130**	19d	7.2	23.6	[4]
**t131**	19e	8.7	22.7	[4]
**t132**	19f	3.9	20.3	[4]
**t133**	19g	80.2	205.9	[4]
**t134**	19h	50.2	58.5	[4]
**t135**	20h	97.5	76.9	[4]
**t136**	21c	56.4	57.6	[4]
**t137**	21f	19.9	42.0	[4]
**t138**	22f	61.0	71.7	[4]
**t139**	26a	10.5	49.8	[4]
**t140**	26b	2.9	19.2	[4]
**t141**	26c	5.2	22.6	[4]
**t142**	26d	21.4	102.0	[4]
**t143**	27b	512.0	456.6	[4]
**t144**	27c	383.0	351.5	[4]
**t145**	28b	27.6	71.1	[4]
**t146**	28c	155.0	242.1	[4]
**t147**	29b	8.4	20.7	[4]
**t148**	29c	7.0	24.6	[4]
**t149**	26e	30.3	21.2	[4]
**t150**	29e	36.4	45.3	[4]

S2. Test set

34 compounds of our research compounds with different substitutions (series **A–H**) were selected in this study

Table S2. Key structure of test set (series **A–H**)

Series	Compound	X	R1	R2
**A**	**5a**	O	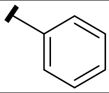	-
	**5b**	O	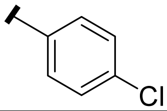	-
	**5c**	S	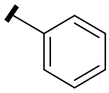	-
**B**	**6a**	-	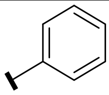	-
	**6b**	-	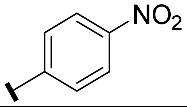	-
	**6c**	-	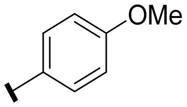	-
**C**	**7a**	-	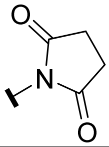	-
	**7b**	-	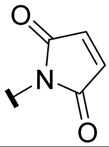	-
	**7c**	-	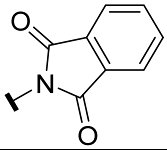	-
**D**	**8a**	-	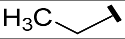	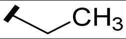
	**8b**	-	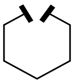	
	**8c**	-	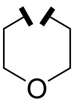	
	**8d**	-	H	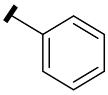
	**8e**	-	H	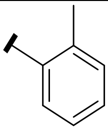

Series	Compound	X	R1	R2
	**8f**	-	H	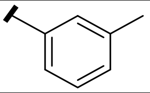
	**8g**	-	H	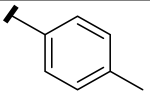
	**28h**	-	H	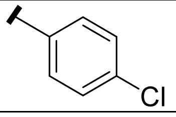
	**8i**	-	H	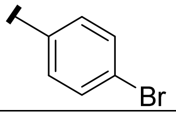
	**8j**	-	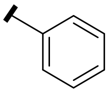	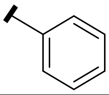
**E**	**9a**	-	H	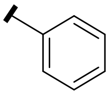
	**9b**	-	H	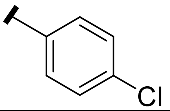
	**9c**	-	H	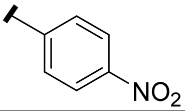
	**9d**	-	H	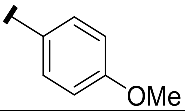
	**9e**	-	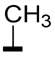	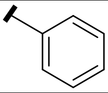
**F**	**10a**	O	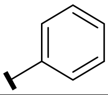	-
	**10b**	O	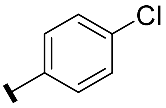	-
	**10c**	S	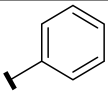	-
**G**	**11a**	-	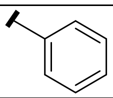	-
	**11b**	-	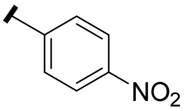	-

Series	Compound	X	R1	R2
	**11c**	-	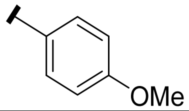	-
**H**	**12a**	-	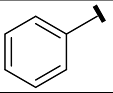	-
	**12b**	-	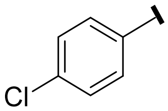	-
	**12c**	-	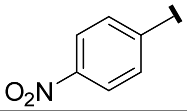	-
	**12e**	-	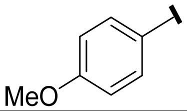	-

S3. Synthetic schemes

S3.1. Scheme S1: Synthetic route of series **A**, **B**, **C**, **F** and **G**.

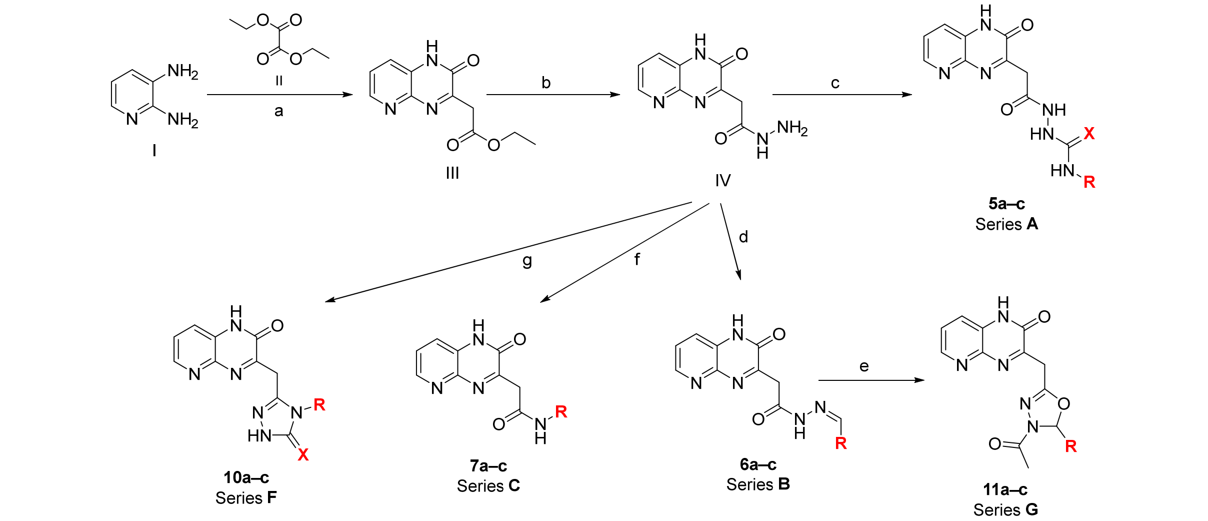

Scheme S1. Reagents and conditions: **a**, Na, EtOH, AcOH, reflux, 30 min.; **b**, hydrazine hydrate, EtOH, reflux, 9 h; **c**, appropriate isocyanate or isothiocyanate, EtOH, reflux, 6 h; **d**, appropriate aromatic aldehyde, AcOH, reflux, 8 h; **e**, acetic anhydride, reflux, 2 h; **f**, appropriate cyclic acid anhydride, AcOH, reflux, 6 h; **g**, appropriate isocyanate or isothiocyanate, EtOH, reflux, 24 h.

S3.2. Scheme S2: Synthetic route of series D, E and H

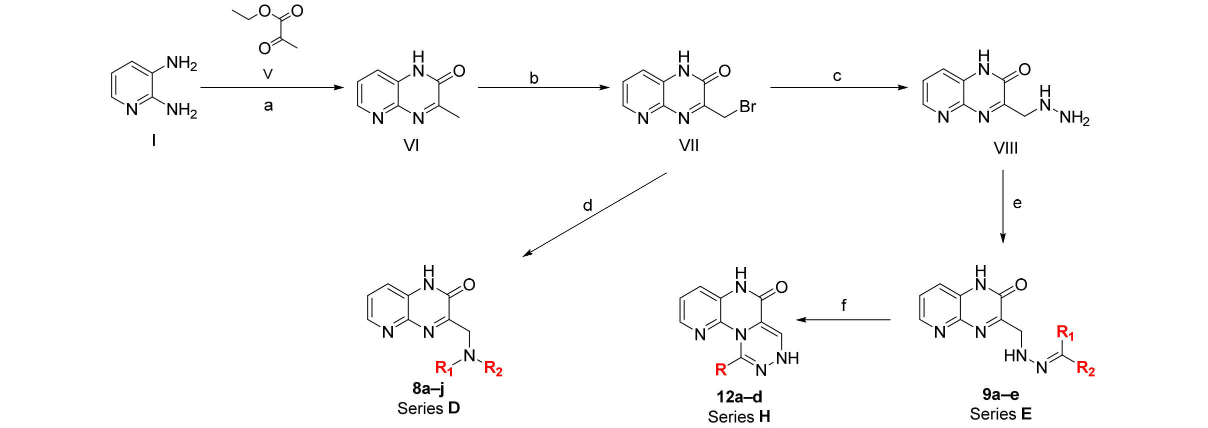

Scheme S2. Synthetic route of series **D**, **E** and **H**. Reagents and conditions: **a**, EtOH, reflux, 10 h; **b**, Br_2, AcOH, NaOAc, reflux, 30 min; **c**, hydrazine hydrate, EtOH, reflux, 9 h; **d**, appropriate amine, EtOH, NaI, reflux, 10 h; **e**, appropriate aldehyde or ketone, AcOH, reflux, 8 h; **f**, Br_2, AcOH, NaOAc, rt, 3 h.

S4. Chemical experimental

Melting points were determined by open capillary tube method using Gallen Kamp melting point apparatus MFB-595-010M (Gallen Kamp, London, England) and were uncorrected. IR Spectra were recorded as potassium bromide discs on Schimadzu FT-IR 8400S spectrophotometer (Shimadzu, Kyoto, Japan) and expressed in wavenumber (ν) cm^{-1}. The ^1H NMR spectra were recorded on *Varian Mercury VX-300 NMR spectrometer at 300 MHz and **Bruker NMR spectrometer at 400 MHz. Chemical Shifts were quoted in δ as parts per million (ppm) downfield from tetramethylsilane (TMS) as internal standard. Mass spectra were recorded using Shimadzu Gas Chromatograph Mass spectrometer QP 1000 Ex (Shimadzu). TLC was carried out using Art. DC-Plastikfolien, Kieselgel $60F^{254}$ sheets (Merck, Darmstadt, Germany). Chloroform/MeOH was used as the developing solvent and the spots were visualized at 366 and 254 nm by UV Vilber Lourmat 77202 (Vilber, Marne La Vallee, France).

S4.1. Synthesis of 3-(ethoxycarbonyl methyl) pyrido[2,3-*b*]pyrazin-2(1*H*)one (III):
Absolute ethanol (25 ml) was added to sodium metal (2.3 g, 0.1 mol). Stand till reaction ceased and then a mixture of ethyl acetate (8.8 g, 9.8 ml, 0.1 ml) and diethyl oxalate (14.6 g, 16.2 ml, 0.1 mol) was slowly added. The mixture was stirred for 30 min. and then added to a solution of 2,3-diaminopyridine (**I**,10.9 g, 0.1 mol) in a mixture of hot ethanol (15 ml) and acetic acid (7 ml). The reaction mixture was heated on a steam bath for 10 min. After cooling, the formed precipitate was filtered and dried. The crude product was crystalized from ethanol to give the titled product **III**. Yield 43%. Mp 234-6 °C. IR $υ_{max}$, cm^{-1}: 3213 (NH), 3100 (CH Ar), 2922, 2850 (CH Aliph), 1697 (2C=O), 1647, 1602, 1562, 1540, 1521 (NH, C=N, C=C). **^1H NMR (400 MHz, DMSO-d_6): δ= 1.23 (t, 3H, $OCH_2\mathbf{CH_3}$), 2.41 (s, 2H, CH_2), 3.89 (q, 2H, $O\mathbf{CH_2}CH_3$), 7.11 (d, *J*= 7.3 Hz, 1H, H-8 Ar), 7.52 (t, 1H, H-7 Ar), 8.11 (d, *J*= 7.0 Hz, 1H, H-6 Ar), 11.49 (s, 1H, NH exchanged with D_2O). Anal. calcd. for $C_{11}H_{11}N_3O_3$ (233.22): C, 56.65; H, 4.75; N, 18.02. Found: C, 56.78; H, 4.88; N, 18.17.

S4.2. Synthesis of 3-hydrazino carbonyl methyl pyrido[2,3-*b*]pyrazin-2(1*H*)one (IV):
Hydrazine hydrate (7.00 g, 6.86 ml, 0.14 mol) was added to a solution of compound **2** (2.33 g, 0.01 mol) in ethanol (20 ml) and refluxed for 9 h. The reaction mixture was cooled in ice bath for 10 min. and filtered, washed with ethanol (5 ml) and dried. The crude product was crystalized from ethanol to give the titled product **IV**. Yield 71%. Mp 214-5 °C. IR $υ_{max}$, cm^{-1}: 3340, 3309 (NH_2, 2NH), 3032 (CH Ar), 2939, 2866 (CH Aliph), 1666 (2C=O), 1620, 1593, 1546, 1512 (NH, C=N, C=C). ^1H NMR (300 MHz, DMSO-d_6): δ= 2.33 (s, 2H, CH_2), 4.61 (s, 2H, NH_2 exchanged with D_2O), 7.21 (d, *J*= 7.1 Hz, 1H, H-8 Ar), 7.51 (t, 1H, H-7 Ar), 8.20 (d, *J*= 6.3 Hz, 1H, H-6 Ar), 10.89 (s, 1H, NH exchanged with D_2O), 11.50 (s, 1H, NH exchanged with D_2O). Anal. calcd. for $C_9H_9N_5O_2$ (219.20): C, 49.31; H, 4.14; N, 31.95. Found: C, 49.11; H, 4.23; N, 31.70.

S4.3. General procedure for synthesis of 3-[4′-((substituted)phenyl (thio)semicarbazido) carbonyl-methyl] pyrido[2,3-*b*]pyrazin-2(1*H*)ones (series A, 5a-c):
Appropriate isocyanate or isothiocyanate (0.3 mol) and compound **IV** (6.57 g, 0.03 mol) were refluxed with ethanol (60 ml) for 6 h. The precipitate was cooled, filtered and dried. The crude product was crystalized from acetic acid.

S4.3.1. 3-[4′-(Phenyl semicarbazido) carbonyl-methyl] pyrido[2,3-*b*]pyrazin-2(1*H*)one (5a):
Yield 55%. Mp 218-20 °C. IR $υ_{max}$, cm^{-1}: 3421, 3375, 3233 (4NH), 3018 (CH Ar), 2951, 2843 (CH Aliph), 1670 (3C=O), 1631, 1593, 1550, 1504 (NH, C=N, C=C). ^1H NMR (300 MHz, DMSO-d_6): δ= 2.21 (s, 2H, CH_2), 5.66 (s, 1H, NH exchanged with D_2O), 6.00 (s, 1H, NH exchanged with D_2O), 7.11 (d, *J*= 7.2 Hz, 1H, H-8 Ar), 7.20-7.45 (m, 6H, H-7,2′,3′,4′,5′,6′ Ar), 8.13 (d, *J*= 8.0 Hz, 1H, H-6 Ar), 10.87 (s, 1H, NH exchanged with D_2O), 11.52 (s, 1H, NH exchanged with D_2O). Anal. calcd. for $C_{16}H_{14}N_6O_3$ (338.32): C, 56.80; H, 4.17; N, 24.84. Found: C, 56.45; H, 4.00; N, 25.01.

S4.3.2. 3-[4′-(4-Chloro phenyl semicarbazido) carbonyl-methyl] pyrido[2,3-*b*]pyrazin-2(1*H*)one (5b)

Yield 54%. Mp 230-2 °C. IR υ_{max}, cm^{-1}: 3417, 3371, 3243 (4NH), 3024 (CH Ar), 2947, 2843 (CH Aliph), 1693 (3C=O), 1631, 1593, 1550, 1500 (NH, C=N, C=C). ^{1}H NMR (300 MHz, DMSO-d_6): δ= 2.26 (s, 2H, CH_2), 5.46 (s, 1H, NH exchanged with D_2O), 6.01 (s, 1H, NH exchanged with D_2O), 7.11 (d, *J*= 7.5 Hz, 1H, H-8 Ar), 7.19-7.51 (m, 5H, H-7,2′,3′,5′,6′ Ar), 8.20 (d, *J*= 6.4 Hz, 1H, H-6 Ar), 10.77 (s, 1H, NH exchanged with D_2O), 11.53 (s, 1H, NH exchanged with D_2O). Anal. calcd. for $C_{16}H_{13}ClN_6O_3$ (372.77): C, 51.55; H, 3.52; N, 22.55. *Found: C, 51.83; H, 3.78; N, 22.62.

S4.3.3. 3-[4′-(Phenyl thiosemicarbazido) carbonyl-methyl] pyrido[2,3-*b*]pyrazin-2(1*H*)one (5c):

Yield 54%. Mp 234-6 °C. IR υ_{max}, cm^{-1}: 3433, 3375, 3275 (4NH), 3028 (CH Ar), 2954, 2843 (CH Aliph), 1693 (2C=O), 1639, 1608, 1593, 1546, 1500 (NH, C=N, C=C), 1265 (C=S). ^{1}H NMR (300 MHz, DMSO-d_6): δ= 2.22 (s, 2H, CH_2), 4.53 (s, 1H, NH exchanged with D_2O), 5.98 (s, 1H, NH exchanged with D_2O), 6.59-6.78 (m, 5H, H-2′,3′,4′,5′,6′ Ar), 7.12 (d, *J*= 7.2 Hz, 1H, H-8 Ar), 7.44 (t, 1H, H-7 Ar), 8.12 (d, *J*= 7.3 Hz, 1H, H-6 Ar), 10.55 (s, 1H, NH exchanged with D_2O), 11.43 (s, 1H, NH exchanged with D_2O). Anal. calcd. for C16H14N6O2S (354.39): C, 54.23; H, 3.98; N, 23.71. *Found: C, 54.52; H, 4.11; N, 23.90.

S4.4. General procedure for synthesis of 3-(*N*^{2}-(substituted)benzylidenehydrazino carbonyl methyl)pyrido[2,3-*b*]pyrazin-2(1*H*)ones (series B, 6a-c):

Appropriate aromatic aldehyde (0.01 mol) was added to a solution of compound **IV** (2.19 g, 0.01 mol) in glacial acetic acid (10 ml) and refluxed for 8 h. The mixture was allowed to cool and poured onto crushed ice (30 g). The formed precipitate was filtered, washed with water (5 ml) and filtered. The crude product was crystalized from ethanol.

S4.4.1. 3-(*N*^{2}-Benzylidenehydrazino carbonyl methyl)pyrido[2,3-*b*]pyrazin2(1*H*)one (6a):

Yield 65%. Mp 228-30 °C. IR υ_{max}, cm^{-1}: 3437, 3371 (2NH), 3018 (CH Ar), 2910, 2824 (CH Aliph), 1678 (2C=O), 1635, 1616, 1604, 1585, 1523 (NH, C=N, C=C). ^{1}H NMR (300 MHz, DMSO-d_6): δ= 2.31 (s, 2H, CH_2), 7.10 (d, *J*= 6.9 Hz, 1H, H-8 Ar), 7.45 (t, 1H, H-7 Ar), 7.77-7.91 (m, 5H, H-2′,3′,4′,5′,6′ Ar), 8.02 (s, 1H, N=CH), 8.30 (d, *J*= 6.1 Hz, 1H, H-6 Ar), 10.96 (s, 1H, NH exchanged with D_2O), 11.61 (s, 1H, NH exchanged with D_2O). Anal. calcd. for $C_{16}H_{13}N_5O_2$ (307.31): C,62.53; H, 4.26; N, 22.79. Found: C, 62.12; H, 4.70; N, 22.41.

S4.4.2. 3-(*N*^{2}-4-Nitro benzylidenehydrazino carbonyl methyl)pyrido[2,3-*b*]pyrazin2(1*H*)one (6b):

Yield 58%. Mp 232-4 °C. IR υ_{max}, cm^{-1}: 3390, 3285 (2NH), 3016 (CH Ar), 2923, 2819 (CH Aliph), 1659 (2C=O), 1628, 1600, 1585, 1573 (NH, C=N, C=C), 1512, 1323(NO_2). ^{1}H NMR (300 MHz, DMSO-d_6): δ= 2.38 (s, 2H, CH_2), 7.03 (d, *J*= 7.2 Hz, 1H, H-8 Ar), 7.40 (t, 1H, H-7 Ar), 7.79 (d, *J*= 7.4 Hz, 2H, H-2′,6′ Ar), 8.11 (s, 1H, N=CH), 8.26-8.40 (m, 3H, H-6,3′,5′ Ar), 10.89 (s, 1H, NH exchanged with D_2O), 11.39 (s, 1H, NH exchanged with D_2O). Anal. calcd. for $C_{16}H_{12}N_6O_4$ (352.30):

S4.4.3. 3-(*N*^{2}-4-Methoxy benzylidenehydrazino carbonyl methyl)pyrido[2,3-*b*]pyrazin2(1*H*)one (6c):

Yield 61%. Mp 222-4 °C. IR υ_{max}, cm^{-1}: 3433, 3213 (2NH), 3032 (CH Ar), 2943, 2843 (CH Aliph), 1697 (2C=O), 1633, 1620, 1612, 1554, 1504 (NH, C=N, C=C). ^{1}H NMR (300 MHz, DMSO-d_6): δ= 2.40 (s, 2H, CH_2), 3.37 (s, 3H, OCH_3), 7.12 (d, *J*= 6.8 Hz, 1H, H-8 Ar), 7.34 (t, 1H, H-7 Ar), 7.50 (m, 4H, H-2′,3′,5′,6′ Ar), 8.09 (s, 1H, N=CH), 8.22 (d, *J*= 5.9 Hz, 1H, H-6 Ar), 10.99 (s, 1H, NH exchanged with D_2O), 11.34 (s, 1H, NH exchanged with D_2O). Anal. calcd. for $C_{17}H_{15}N_5O_3$ (337.33): C, 60.53; H, 4.48; N, 20.76. Found: C, 60.33; H, 4.73; N, 20.98.

S4.5. General procedure for synthesis of 3-[*N*-(substituted)carbamoyl methyl]pyrido[2,3-*b*]pyrazin-2(1*H*)ones (series C, 7a-c):

Appropriate cyclic acid anhydrides (0.005 mol) were added to solution of compound **IV** (1.1 g, 0.005 mol) in glacial acetic acid (10 ml) and refluxed for 6 h. After cooling, the mixture was poured onto crushed ice (30 g). The formed precipitate was filtered, washed with water (5 ml) and dried. The crude product was crystalized from ethanol.

S4.5.1. 3-[*N*-(Succinimido)carbamoyl methyl]pyrido[2,3-*b*]pyrazin-2(1*H*)one (7a):
Yield 82%. Mp 210-12 °C. IR υ_{max}, cm^{-1}: 3426, 3410 (2NH), 3063 (CH Ar), 2939, 2866 (CH Aliph), 1681 (4C=O), 1635, 1629, 1554, 1516 (NH, C=N, C=C). ^{1}H NMR (300 MHz, DMSO-*d*$_6$): δ= 2.12 (s, 2H, CH_2), 2.78 (t, 4H, **C**$\mathbf{H_2}$**C**$\mathbf{H_2}$), 7.12 (d, *J*= 7.3 Hz, 1H, H-8 Ar), 7.41 (t, 1H, H-7 Ar), 8.12 (d, *J*= 7.3 Hz, 1H, H-6 Ar), 10.49 (s, 1H, NH exchanged with D_2O), 11.50 (s, 1H, NH exchanged with D_2O). Anal. calcd. for $C_{13}H_{11}N_5O_4$ (301.26): C, 51.83; H, 3.68; N, 23.25. Found: C, 52.21; H, 3.80; N, 23.13.

S4.5.2. 3-[*N*-(Maleic imido)carbamoyl methyl]pyrido[2,3-*b*]pyrazin-2(1*H*)one (7b):
Yield 85%. Mp 213-5 °C. IR υ_{max}, cm^{-1}: 3341, 3290 (2NH), 3036 (CH Ar), 2947, 2866 (CH Aliph), 1659 (4C=O), 1647, 1620, 1600, 1585 (NH, C=N, C=C). ^{1}H NMR (300 MHz, DMSO-*d*$_6$): δ= 2.22 (s, 2H, CH_2), 5.54 (d, *J*= 7.8 Hz, 2H, C**H**=C**H**), 7.21 (d, *J*= 7.2 Hz, 1H, H-8 Ar), 7.44 (t, 1H, H-7 Ar), 8.10 (d, *J*= 7.0 Hz, 1H, H-6 Ar), 10.22 (s, 1H, NH exchanged with D_2O), 11.33 (s, 1H, NH exchanged with D_2O). Anal. calcd. For $C_{13}H_9N_5O_4$ (299.24): C, 52; H, 3.03; N, 23.40. Found: C, 51.92; H, 3.33; N, 23.76.

S4.5.3. 3-[*N*-(Phthalimido)carbamoyl methyl]pyrido[2,3-*b*]pyrazin-2(1*H*)one (7c):
Yield 78%. Mp 211-13 °C. IR υ_{max}, cm^{-1}: 3429, 3356 (2NH), 3020 (CH Ar), 2939, 2858 (CH Aliph), 1670 (4C=O), 1620, 1600, 1590, 1500 (NH, C=N, C=C). ^{1}H NMR (300 MHz, DMSO-*d*$_6$): δ= 2.20 (s, 2H, CH_2), 7.11 (d, *J*= 7.0 Hz, 1H, H-8 Ar), 7.34 (t, 1H, H-7 Ar), 7.66-798 (m, 4H, H-3′,4′,5′,6′ Ar), 8.21 (d, *J*= 7.2 Hz, 1H, H-6 Ar), 10.41 (s, 1H, NH exchanged with D_2O), 11.55 (s, 1H, NH exchanged with D_2O). Anal. calcd. for $C_{17}H_{11}N_5O_4$ (349.30): C, 58.45; H, 3.17; N, 20.05. Found: C, 58.12; H, 3.19; N, 19.80.

S4.6. General procedure for synthesis of 3-[4′-((substituted)phenyl)-5′ (thio)oxo-4′*H*-1′,2′,4′-triazol-3′-yl)methyl]pyrido[2,3-*b*]pyrazin-2(1*H*)ones (series F, 10a-c):
Appropriate isocyanate or isothiocyanate (0.12 mol) and compound **IV** (0.22 g, 0.001 mol) were refluxed with ethanol (50 ml) for 24 h. The precipitate was cooled, filtered and dried. The crude product was crystalized from acetic acid.

S4.6.1. 3-[4′-(Phenyl)-5′ oxo-4′*H*-1′,2′,4′-triazol-3′-yl)methyl]pyrido[2,3-*b*]pyrazin-2(1*H*)one (10a):
Yield 77%. Mp 220-2 °C. IR υ_{max}, cm^{-1}: 3422, 3375 (2NH), 3082 (CH Ar), 2951, 2843 (CH Aliph), 1689 (2C=O), 1647, 1593, 1546, 1500 (NH, C=N, C=C). ^{1}H NMR (300 MHz, DMSO-*d*$_6$): δ= 2.34 (s, 2H, CH_2), 4.31 (s, 1H, NH exchanged with D_2O), 7.11 (d, *J*= 8.0 Hz, 1H, H-8 Ar), 7.43 (t, 1H, H-7 Ar), 7.60-8.10 (m, 5H, H-2′,3′,4′,5′,6′ Ar), 8.21 (d, *J*= 7.1 Hz, 1H, H-6 Ar), 11.39 (s, 1H, NH exchanged with D_2O). Anal. calcd. for $C_{16}H_{12}N_6O_2$ (320.31): C, 60.00; H, 3.78; N, 26.24. Found: C, 60.23; H, 4.01; N, 26.54.

S4.6.2. 3-[4′-(4-Chloro phenyl)-5′ oxo-4′*H*-1′,2′,4′-triazol-3′-yl)methyl]pyrido[2,3-*b*]pyrazin-2(1*H*)one (10b):
Yield 68%. Mp 228-30 °C. IR υ_{max}, cm^{-1}: 3444, 3275 (2NH), 3032 (CH Ar), 2954, 2845 (CH Aliph), 1689 (2C=O), 1647, 1589, 1546, 1500 (NH, C=N, C=C). ^{1}H NMR (300 MHz, DMSO-*d*$_6$): δ= 2.31 (s, 2H, CH_2), 4.22 (s, 1H, NH exchanged with D_2O), 7.11 (d, *J*= 7.4 Hz, 1H, H-8 Ar), 7.34 (t, 1H, H-7 Ar), 7.55-7.91 (m, 4H, H-2′,3′,5′,6′ Ar), 8.26 (d, *J*= 7.3 Hz, 1H, H-6 Ar), 11.45 (s, 1H, NH exchanged with D_2O). Anal. calcd. for $C_{16}H_{11}ClN_6O_2$ (354.75): C, 54.17; H, 3.13; N, 23.69. *Found: C, 54.32; H, 3.22; N, 24.00.

S4.6.3. 3-[4′-(Phenyl)-5′ thioxo-4′*H*-1′,2′,4′-triazol-3′-yl)methyl]pyrido[2,3-*b*]pyrazin-2(1*H*)one (10c):
Yield 71%. Mp 219-22 °C. IR υ_{max}, cm^{-1}: 3444, 3275 (2NH), 3032 (CH Ar), 2954, 2846 (CH Aliph), 1689 (C=O), 1647, 1593, 1546, 1500 (NH, C=N, C=C), 1270 (C=S). ^{1}H NMR (300 MHz, DMSO-d_6): δ= 2.28 (s, 2H, CH_2), 4.00 (s, 1H, NH exchanged with D_2O), 6.60-7.11 (m, 5H, H-2′,3′,4′,5′,6′ Ar), 7.21 (d, *J*= 7.2 Hz, 1H, H-8 Ar), 7.44 (t, 1H, H-7 Ar), 8.22 (d, *J*= 6.0 Hz, 1H, H-6 Ar), 11.51 (s, 1H, NH exchanged with D_2O). Anal. calcd. for $C_{16}H_{12}N_6OS$ (336.37): C, 57.13; H, 3.60; N, 24.98. *Found: C, 57.22; H, 3.61; N, 24.67.

S4.7. General procedure for synthesis of 3-[(3′-acetyl-2′- (substituted)phenyl-2′-3′-dihydro-1′,3′,4′-oxadiazolo-5′-yl) methyl[2,3-*b*]pyrazin-2(1*H*)ones (series G, 11a-c):
A mixture of compounds **6a-c** (0.01 mol) and acetic anhydride (6 ml) were refluxed for 2 h. the excess acetic anhydride was distilled off under reduced pressure. The obtained residue was triturated with petroleum ether (25 ml). The precipitate was filtered and dried. The crude product was crystalized from benzene.

S4.7.1. 3-[(3′-Acetyl-2′- phenyl-2′-3′-dihydro-1′,3′,4′-oxadiazolo-5′-yl) methyl[2,3-*b*]pyrazin-2(1*H*)one (11a):
Yield 53%. Mp 234-6 °C. IR υ_{max}, cm^{-1}: 3444 (NH), 3035 (CH Ar), 2947, 2846 (CH Aliph), 1693 (2C=O), 1639, 1627, 1589, 1523 (NH, C=N, C=C). ^{1}H NMR (300 MHz, DMSO-d_6): δ= 2.13 (s, 2H, CH_2), 2.34 (s, 3H, $COCH_3$), 6.23 (s, 1H, CH oxadiazole), 7.68-7.09 (m, 5H, H-2′,3′,4′,5′,6′ Ar), 7.13 (d, *J*= 7.1 Hz, 1H, H-8 Ar), 7.33 (t, 1H, H-7 Ar), 8.21 (d, *J*= 6.2 Hz, 1H, H-6 Ar), 11.51 (s, 1H, NH exchanged with D_2O). Anal. calcd. for $C_{18}H_{15}N_5O_3$ (349.34): C, 61.89; H, 4.33; N, 20.05. Found: C, 62.03; H, 4.11; N, 19.76.

S4.7.2. 3-[(3′-Acetyl-2′-(4-nitro phenyl)-2′-3′-dihydro-1′,3′,4′-oxadiazolo-5′-yl) methyl[2,3-*b*]pyrazin-2(1*H*)one (11b):
Yield 55%. Mp 240-2 °C. IR υ_{max}, cm^{-1}: 3337 (NH), 3043 (CH Ar), 2951, 2854 (CH Aliph), 1651 (2C=O), 1635, 1600, 1554, 1532 (NH, C=N, C=C), 1500, 1335 (NO_2). ^{1}H NMR (300 MHz, DMSO-d_6): δ= 2.02 (s, 2H, CH_2), 2.33 (s, 3H, $COCH_3$), 6.50 (s, 1H, CH oxadiazole), 7.12 (d, *J*= 6.8 Hz, 1H, H-8 Ar), 7.34 (t, 1H, H-7 Ar), 7.41-7.80 (m, 4H, H-2′,3′,5′,6′ Ar), 8.13 (d, *J*= 5.8 Hz, 1H, H-6 Ar), 11.48 (s, 1H, NH exchanged with D_2O). Anal. calcd. for $C_{18}H_{14}N_6O_5$ (394.34): C, 54.82; H, 3.58, N, 21.31. *Found: C, 54.45; H, 3.42; N, 21.09.

S4.7.3. 3-[(3′-Acetyl-2′-(4-methoxy phenyl)-2′-3′-dihydro-1′,3′,4′-oxadiazolo-5′-yl) methyl[2,3-*b*]pyrazin-2(1*H*)one (11c):
Yield 47%. Mp 235-7 °C. IR υ_{max}, cm^{-1}: 3441 (NH), 3067 (CH Ar), 2931, 2843 (CH Aliph), 1693 (2C=O), 1627, 1600, 1551, 1508 (NH, C=N, C=C). ^{1}H NMR (300 MHz, DMSO-d_6): δ= 2.13 (s, 2H, CH_2), 2.34 (s, 3H, $COCH_3$), 3.65 (s, 3H, OCH_3), 6.71 (s, 1H, CH oxadiazole), 6.82-7.01 (m, 4H, H-2′,3′,5′,6′ Ar), 7.21 (d, *J*= 7.6 Hz, 1H, H-8 Ar), 7.42 (t, 1H, H-7 Ar), 8.23 (d, *J*= 6.3 Hz, 1H, H-6 Ar), 11.39 (s, 1H, NH exchanged with D_2O). MS: *m*/*z*% 379, M^+, (1.92%). Anal. calcd. for $C_{19}H_{17}N_5O_4$ (379.37): C, 60.15; H, 4.52; N, 18.46. *Found: C, 60.51; H, 4.69; N, 18.80.

S4.8. 3-Methylpyrido[2,3-b]pyrazin-2(1H)one (VI):
Ethyl pyruvate (**V**, 2.90 g, 2.77 ml, 0.025 mol) was added to a solution of 2,3-diaminopyridine (**I**, 2.75 g, 0.025 mol) in ethanol (30 ml) and refluxed for 10 h. Reaction mixture was cooled, filtered, washed with ethanol (5 ml) and dried. The crude product was crystalized from ethanol. Yield 59%, mp 278-280 °C, IR (KBr) [υ /cm^{-1}]: 3186 (NH), 3018 (CH Ar), 2943, 2839 (CH Aliph), 1685 (C=O), 1641, 1631, 1597, 1559, 1546, 1527 (C=N, NH, C=C). ^{1}HNMR *(300 MHz, DMSO-d_6): δ= 2.41 (s, 3H, CH_3), 7.48 (d, *J*= 8.4 Hz, 1H, H-8Ar), 7.67 (t, 1H, H-7Ar), 8.11 (d, *J*= 7.6 Hz, 1H, H-6Ar)), 12.44 (s, 1H, NH, exchanged with D_2O). Anal. Calcd. for $C_8H_7N_3O$ (161.16): C, 59.62; H, 4.38; N, 26.07. Found: C, 59.90; H, 4.14; N, 26.73.

S4.9. 3-Bromomethylpyrido[2,3-b]pyrazin-2(1H)one (VII):
Compound **VI** (3.22g, 0.02 mol) was dissolved in glacial acetic acid (10 ml) and anhydrous sodium acetate (1.64 g, 0.02 mol) was added. Bromine (3.04 g, 0.99 ml, 0.019 mol) was added dropwise and the mixture was heated on steam bath for 30 min. then cooled. The formed precipitate was filtered, washed with glacial acetic acid (5 ml) and dried. The crude product was crystalized from ethanol. Yield 43%, mp 237-239°C, IR (KBr) [υ /cm^{-1}]: 3414 (NH), 3055 (CH Ar), 2980, 2883 (CH Aliph), 1670 (C=O), 1647, 1616, 1558, 1544, 1508 (C=N, NH, C=C). ^{1}HNMR *(300 MHz, DMSO-*d$_6$*): δ= 3.10 (s, 2H, CH_2), 7.40 (d, *J*= 7.4 Hz, 1H, H-8Ar), 7.61 (t, 1H, H-7Ar), 8.35 (d, *J*= 6.1 Hz, 1H, H-6Ar), 11.50 (s, 1H, NH, exchanged with D_2O). MS (*m/z*) %; 240 (M^+) 0.53%, 242 (M^++2) 0.67%. Anal. Calcd. for $C_8H_6BrN_3O$ (240.06): C, 40.03; H, 2.52; N, 17.50. Found: C, 40.13; H, 2.47; N, 17.68.

S4.10. General procedure for synthesis of 3-substituted methylpyrido[2,3-b]pyrazin-2(1H)ones (series D, 8a-j):
Appropriate amine (0.02 mol) was added to a solution of compound **VII** (2.4 g, 0.01 mol) in ethanol (30 ml) containing sodium iodide (0.15 g, 0.001 mol) and refluxed for 10 h. The reaction mixture was cooled, formed precipitate filtered, washed with ethanol (5 ml) and dried. The crude product was crystalized from ethanol.

S4.10.1. 3-(N,N-Diethylamino)methylpyrido[2,3-b]pyrazin-2(1H)one (8a):
Yield 81%, mp 262-263°C, IR (KBr) [υ/cm^{-1}]: 3238 (NH), 3051 (CH Ar), 2924, 2852 (CH Aliph), 1672 (C=O), 1637, 1624, 1612, 1593, 1508 (C=N, NH, C=C). ^{1}HNMR *(300 MHz, DMSO-*d$_6$*): δ= 1.41 (t, 6H, 2x CH_2**CH_3**), 2.29 (q, 4H, 2x **CH_2**CH_3), 2.43 (s, 2H, CH_2), 7.11 (d, *J*= 7.4 Hz, 1H, H-8Ar), 7.30 (t, 1H, H-7Ar), 8.21 (d, *J*= 6.4 Hz, 1H, H-6Ar), 10.80 (s, 1H, NH, exchanged with D_2O). Anal. Calcd. for $C_{12}H_{16}N_4O$ (232.28): C, 62.05; H, 6.94; N, 24.12. Found: C, 62.18; H, 6.97; N, 24.13.

S4.10.2. 3-(Piperidin-1-yl))methylpyrido[2,3-b]pyrazin-2(1H)one (8b):
Yield 80%, mp 277-279 °C, IR (KBr) [υ/cm^{-1}]: 3387 (NH), 3066 (CH Ar), 2964, 2866 (CH Aliph), 1700 (C=O), 1649, 1612, 1591, 1508, 1500 (C=N, NH, C=C). ^{1}HNMR (400 MHz, DMSO-*d$_6$*): δ= 2.06-2.32 (m, 10H, piperidine H), 2.51 (s, 2H, CH_2), 7.31 (d, *J*=8.0 Hz, 1H, H-8Ar), 7.53 (t, 1H, H-7 Ar), 8.22 (d, *J*= 6.6 Hz, 1H, H-6 Ar), 11.13 (s, 1H, NH, exchanged with D_2O). Anal. Calcd. for $C_{13}H_{16}N_4O$ (244.29): C, 63.91; H, 6.60; N, 22.93. Found: C, 63.98; H, 6.64; N, 23.10.

S4.10.3. 3-(Morpholin-4-yl)methylpyrido[2,3-b]pyrazin-2(1H)one (8c):
Yield 74%, mp 234-235 °C, IR (KBr) [υ/cm^{-1}]: 3394 (NH), 3059 (CH Ar), 2920, 2840 (CH Aliph), 1674 (C=O), 1640, 1618, 1593, 1580, 1540 (C=N, NH, C=C). ^{1}HNMR *(300 MHz, DMSO-*d$_6$*): δ= 2.04 (t, 4H, morpholine H), 2.41 (s, 2H, CH_2), 3.30 (t, 4H, morpholine H), 7.33 (d, *J*= 8.0 Hz, 1H, H-8 Ar), 7.75 (t, 1H, H-7 Ar), 8.12 (d, *J*= 7.4 Hz, 1H, H-6 Ar), 10.89 (s, NH, exchanged with D_2O). Anal. Calcd. for $C_{12}H_{14}N_4O_2$ (246.27): C, 58.53; H, 5.73; N, 22.75. Found: C, 58.61; H, 5.71; N, 22.92.

S4.10.4. 3-Phenylaminomethylpyrido[2,3-b]pyrazin-2(1H)one (8d):
Yield 60%, mp 248-250 °C, IR (KBr) [υ/cm^{-1}]: 3404, 3385 (2NH), 3049 (CH Ar), 2926, 2852 (CH Aliph), 1672 (C=O), 1593, 1560, 1521, 1508 (C=N, NH, C=C). ^{1}HNMR (400 MHz, DMSO-*d$_6$*): δ= 3.10 (s, 2H, CH_2), 6.52-7.10 (m, 5H, H-2′, 3′, 4′, 5′, 6′ Ar), 7.21 (d, *J*= 9.6 Hz, 1H, H-8Ar), 7.50 (t, 1H, H-7Ar), 8.17 (d, *J*= 8.2 Hz, 1H, H-6Ar), 10.62 (s, 1H, NH, exchanged with D_2O), 11.51 (s, 1H, NH, exchanged with D_2O). Anal. Calcd. for $C_{14}H_{12}N_4O$ (252.27): C, 66.65; H, 4.79; N, 22.21. Found: C, 66.83; H, 4.74; N, 22.37.

S4.10.5. 3-(2-Methylphenyl)aminomethylpyrido[2,3-b]pyrazin-2(1H)one (8e):

Yield 64%, mp 296-298°C, IR (KBr) [ʋ/cm^{-1}]: 3414, 3385 (2NH), 3049 (CH Ar), 2924, 2854 (CH Aliph), 1672 (C=O), 1649, 1622, 1593, 1570, 1521 (C=N, NH, C=C). ^{1}HNMR (400 MHz, DMSO-d_6): δ= 2.13 (s, 3H, CH_3), 3.11 (s, 2H, CH_2), 6.30-7.00 (m, 4H, H-3′, 4′, 5′, 6′ Ar), 7.21 (d, *J*= 7.2 Hz, 1H, H-8Ar), 7.50 (t, 1H, H-7Ar), 8.16 (d, *J*= 8.4 Hz, 1H, H-6Ar), 10.80 (s, 1H, NH, exchanged with D_2O), 11.44 (s, 1H, NH, exchanged with D_2O). Anal. Calcd. for $C_{15}H_{14}N_4O$ (266.30): C, 67.65; H, 5.30; N, 21.04. Found: C, 67.82; H, 5.32; N, 21.08.

S4.10.6. 3-(3-Methylphenyl)aminomethylpyrido[2,3-b]pyrazin-2(1H)one (8f):

Yield 57%, mp 258-260 °C, IR (KBr) [ʋ/cm^{-1}]: 3414, 3388 (2NH), 3057 (CH Ar), 2922, 2852 (CH Aliph), 1674 (C=O), 1622, 1593, 1558, 1518 (C=N, NH, C=C). ^{1}HNMR (400 MHz, DMSO-d_6): δ= 2.15 (s, 3H, CH_3), 3.32 (s, 2H, CH_2), 6.12-6.99 (m, 4H, H-2′, 4′, 5′, 6′ Ar), 7.20 (d, *J*= 6.3 Hz, 1H, H-8 Ar), 7.50 (t, 1H, H-7 Ar), 8.19 (d, *J*= 8.3 Hz, 1H, H-6 Ar), 10.81 (s, 1H, NH, exchanged with D_2O), 11.50 (s, 1H, H NH, exchanged with D_2O). Anal. Calcd. for $C_{15}H_{14}N_4O$ (266.30): C, 67.65; H, 5.30; N, 21.04. Found: C, 67.84; H, 5.34;N, 21.06.

S4.10.7. 3-(4-Methylphenyl)aminomethylpyrido[2,3-b]pyrazin-2(1H)one (8g):

Yield 73%, mp 284-286 °C, IR (KBr) [ʋ/cm^{-1}]: 3369, 3350 (2NH), 3051 (CH Ar), 2926, 2856 (CH Aliph), 1672 (C=O), 1612, 1591, 1544, 1514 (C=N, NH, C=C). ^{1}HNMR *(300 MHz, DMSO-d_6): δ= 2.10 (s, 3H, CH_3), 2.41 (s, 2H, CH_2), 6.30-6.83 (m, 4H, H-2′, 3′, 5′, 6′ Ar), 7.22 (d, *J*= 6.7 Hz, 1H, H-8 Ar), 7.54 (t, 1H, H-7 Ar), 8.17 (d, *J*= 7.0 Hz, 1H, H-6 Ar), 10.09 (s, 1H, NH, exchanged with D_2O), 11.51 (s, 1H, NH, exchanged with D_2O). Anal. Calcd. for $C_{15}H_{14}N_4O$ (266.30): C, 67.65; H, 5.30; N, 21.04. Found: C, 67.80; H, 5.30;N, 21.18.

S4.10.8. 3-(4-Chlorophenyl)aminomethylpyrido[2,3-b]pyrazin-2(1H)one (8h):

Yield 66%, mp>300 °C, IR (KBr) [ʋ/cm^{-1}]: 3421, 3385 (2NH), 3093 (CH Ar), 2924, 2852 (CH Aliph), 1674 (C=O), 1647, 1624, 1593, 1570 (C=N, NH, C=C). ^{1}HNMR (400 MHz, DMSO-d_6): δ= 3.31 (s, 2H, CH_2), 6.42-7.12 (m, 4H, H-2′, 3′, 5′, 6′ Ar), 7.21 (d, *J*= 6.3 Hz, 1H, H-8 Ar), 7.59 (t, 1H, H-7 Ar), 8.11 (d, *J*= 6.2 Hz, 1H, H-6 Ar), 11.00 (s, 1H, NH, exchanged with D_2O), 11.59 (s, 1H, NH, exchanged with D_2O). Anal. Calcd. for $C_{14}H_{11}ClN_4O$ (286.72): C, 58.65; H, 3.87; N, 19.54. Found: C, 58.78; H, 3.92; N, 19.73.

S4.10.9. 3-(4-Bromophenyl)aminomethylpyrido[2,3-b]pyrazin-2(1H)one (8i):

Yield 79%, mp>300 °C, IR (KBr) [ʋ/cm^{-1}]: 3400, 3388 (2NH), 3101 (CH Ar), 2924, 2897 (CH Aliph), 1664 (C=O), 1649, 1618, 1593 (C=N, NH, C=C). ^{1}HNMR (400 MHz, DMSO-d_6): δ= 3.11 (s, 2H, CH_2), 6.36-7.20 (m, 4H, H-2′, 3′, 5′, 6′ Ar), 7.41 (d, *J*= 8.1 Hz, 1H, H-8 Ar), 7.69 (t, 1H, H-7 Ar), 8.22 (d, *J*= 6.0 Hz, 1H, H-6 Ar), 11.02 (s, 1H, NH, exchanged with D_2O), 11.61 (s, 1H, NH, exchanged with D_2O). Anal. Calcd. for $C_{14}H_{11}BrN_4O$ (331.17): C, 50.77; H, 3.35; N, 16.92. Found: C, 50.91; H, 3.37; N, 16.98.

S4.10.10. 3-(N,N-Diphenylamino)methylpyrido[2,3-b]pyrazin-2(1H)one (8j):

Yield 46%, mp 292-293°C, IR (KBr) [ʋ/cm^{-1}]: 3410 (NH), 3057 (CH Ar), 2924, 2852 (CH Aliph), 1662 (C=O), 1649, 1610, 1591, 1570, 1508 (C=N, NH, C=C). ^{1}HNMR (400 MHz, DMSO-d_6): δ= 3.32 (s, 2H, CH_2), 6.50-7.11 (m, 10H, Ar H), 7.21 (d, *J*= 7.2 Hz, 1H, H-8 Ar), 7.55 (t, 1H, H-7 Ar), 8.15 (d, *J*= 7.0 Hz, 1H, H-6 Ar), 11.44 (s, 1H, NH, exchanged with D_2O). Anal. Calcd. for $C_{20}H_{16}N_4O$ (328.37): C, 73.15; H, 4.91; N, 17.06. Found: C, 73.28; H, 4.97; N, 17.22.

S4.11. 3-Hydrazinomethylpyrido[2,3-b]pyrazin-2(1H)one (VIII):

Hydrazine hydrate (7.00 g, 6.86 ml, 0.14 mol) was added to a solution of compound **VII** (2.40 g, 0.01 mol) in ethanol (20 ml) and refluxed for 9 h. The mixture was cooled in ice bath for 10 min. and formed precipitate was filtered. The precipitate was washed with ethanol (5 ml) and dried. The crude product was crystallized from ethanol. Yield 73%, mp 243-245 °C, IR (KBr) [ʋ/cm^{-1}]: 3369, 3329 (NH_2, 2NH), 3066 (CH Ar), 2924, 2852 (CH Aliph), 1670 (C=O), 1612, 1591, 1570, 1508 (C=N, NH, C=C). ^{1}HNMR (400 MHz, DMSO-

*d*6): δ= 2.51 (s, 2H, CH_2), 7.21 (d, *J*= 6.2 Hz, 1H, H-8 Ar), 7.50 (t, 1H, H-7 Ar), 8.22 (d, *J*= 8.2 Hz, 1H, H-6Ar), 10.72 (s, 1H, NH, exchanged with D_2O), 11.22 (s, 2H, NH_2, exchanged with D_2O), 11.50 (s, 1H, NH, exchanged with D_2O). Anal. Calcd. for $C_8H_9N_5O$ (191.19): C, 50.26; H, 4.74; N, 36.63. Found: C, 50.41; H, 4.73; N, 37.01.

S4.12. General procedure for synthesis of 3-(2-(un)substituted benzylidene)hydrazinomethylpyrido[2,3-b]pyrazin-2(1H)-ones (series E, 9a-e):

Appropriate aromatic aldehyde or ketone (0.01 mol) was added to a solution of compound **VIII** (1.91 g, 0.01 mol) in glacial acetic acid (10 ml) and refluxed for 8 h. The mixture was allowed to cool then poured onto crushed ice (30 g). The precipitate was filtered, washed with water (5 ml) and dried. The crude product was crystallized from ethanol.

S4.12.1. 3-(2-Benzylidene)hydrazinomethylpyrido[2,3-b]pyrazin-2(1H)-one (9a):

Yield 62%, mp 234-236°C, IR (KBr) [ʋ/cm^{-1}]: 3398, 3364 (2NH), 3061 (CH Ar), 2901, 2829 (CH Aliph), 1676 (C=O), 1624, 1600, 1583, 1544, 1508 (C=N, NH, C=C). ^{1}HNMR (400 MHz, DMSO-*d*6): δ= 2.32 (s, 2H, CH_2), 7.21 (d, *J*= 7.2 Hz, 1H, H-8 Ar), 7.31-7.42 (m, 3H, H-3′,4′,5′ Ar), 7.45 (t, 1H, H-7 Ar), 7.62 (d, *J*= 6.7 Hz, 2H, H-2′,6′ Ar), 8.11 (s, 1H, N=CH), 8.22 (d, *J*= 6.2 Hz, 1H, H-6 Ar), 11.32 (s, 1H, NH, exchanged with D_2O), 11.54 (s, 1H, NH, exchanged with D_2O). Anal. Calcd. for $C_{15}H_{13}N_5O$ (279.30): C, 64.51; H, 4.69; N, 25.07. Found: C, 64.67; H, 4.73; N, 25.19.

S4.12.2. 3-[2-(4-Chlorobenzylidene)]hydrazinomethylpyrido[2,3-b]pyrazin-2(1H)-one (9b):

Yield 69%, mp 237-239°C, IR (KBr) [ʋ/cm^{-1}]: 3421, 3387 (2NH), 3012 (CH Ar), 2920, 2837 (CH Aliph), 1683 (C=O), 1616, 1593, 1558, 1541, 1521 (C=N, NH, C=C). ^{1}HNMR (400 MHz, DMSO-*d*6): δ= 2.42 (s, 2H, CH_2), 7.22 (d, *J*= 7.3 Hz, 1H, H-8 Ar), 7.34 (d, *J*= 6.1 Hz, 2H, H-3′,5′ Ar), 7.46 (t, 1H, H-7 Ar), 7.59 (d, *J*= 7.0 Hz, 2H, H-2′,6′ Ar), 8.11 (s, 1H, N=CH), 8.22 (d, *J*= 6.3 Hz, 1H, H-6 Ar), 11.30 (s, 1H, NH, exchanged with D_2O), 11.51 (s, 1H, NH, exchanged with D_2O). MS (*m*/*z*)%; 313 (M^+) 2.63%, 315 (M^++2) 2.77%. Anal. Calcd. for $C_{15}H_{12}ClN_5O$ (313.74): C, 57.42; H, 3.86; N, 22.32. Found: C, 57.52; H, 3.91;N, 22.48.

S4.12.3. 3-[2-(4-Nitrobenzylidene)]hydrazinomethylpyrido[2,3-b]pyrazin-2(1H)-one (9c):

Yield 70%, mp 238-240 °C, IR (KBr) [ʋ/cm^{-1}]: 3444, 3365 (2NH), 3021 (CH Ar), 2926, 2850 (CH Aliph), 1677 (C=O), 1653, 1614, 1597, 1558 (C=N, NH, C=C), 1519, 1344 (NO_2). ^{1}HNMR (400 MHz, DMSO-*d*6): δ= 2.33 (s, 2H, CH_2), 7.21 (d, *J*= 6.4 Hz, 1H, H-8 Ar), 7.66 (t, 1H, H-7 Ar), 7.91-8.10 (m, 4H, H-2′, 3′, 5′, 6′ Ar), 8.19 (s, 1H, N=CH), 8.23 (d, *J*= 7.2 Hz, 1H, H-6 Ar), 11.32 (s, 1H, NH, exchanged with D_2O), 11.50 (s, 1H, NH, exchanged with D_2O). Anal. Calcd. for $C_{15}H_{12}N_6O_3$ (324.29): C, 55.55; H, 3.73; N, 25.91. Found: C, 55.74; H, 3.74; N, 26.07.

S4.12.4. 3-[2-(4-Methoxybenzylidene)]hydrazinomethylpyrido[2,3-b]pyrazin-2(1H)-one (9d):

Yield 63%, mp 256-258 °C, IR (KBr) [ʋ/cm^{-1}]: 3417, 3392 (2NH), 3039 (CH Ar), 2933, 2837 (CH Aliph), 1670 (C=O), 1598, 1575, 1558, 1541, 1508 (C=N, NH, C=C). ^{1}HNMR *(300 MHz, DMSO-*d*6): δ= 2.12 (s, 2H, CH_2), 3.52 (s, 3H, OCH_3), 6.76 (d, *J*= 8.2 Hz, 2H, H-3′,5′ Ar), 7.21 (d, *J*= 7.1 Hz, 1H, H-8 Ar), 7.43 (t, 1H, H-7 Ar), 7.66 (d, *J*= 7.4 Hz, 2H, H-2′,6′ Ar), 8.10 (s, 1H, N=CH), 8.22 (d, *J*= 7.6 Hz, 1H, H-6 Ar), 11.30 (s, 1H, NH, exchanged with D_2O), 11.45 (s, 1H, NH, exchanged with D_2O). Anal. Calcd. for $C_{16}H_{15}N_5O_2$ (309.32): C, 62.13; H, 4.89; N, 22.64. Found: C, 62.26; H, 4.87; N, 22.72.

S4.12.5. 3-(2-Methyl-2-phenyl)hydrazinomethylpyrido[2,3-b]pyrazin-2(1H)-one (9e):

Yield 69%, mp 230-233°C, IR (KBr) [ʋ/cm^{-1}]: 3446, 3363 (2NH), 3032 (CH Ar), 2929, 2856 (CH Aliph), 1670 (C=O), 1645, 1616, 1570, 1558, 1521, (C=N, NH, C=C). ^{1}HNMR (400 MHz, DMSO-*d*6): δ= 1.42 (s, 3H, CH_3),

2.50 (s, 2H, CH_2), 7.22 (d, *J*= 6.2 Hz, 1H, H-8 Ar), 7.30-7.43 (m, 3H, H-3′,4′,5′ Ar), 7.49 (t, 1H, H-7 Ar), 7.63 (d, *J*= 8.1 Hz, 2H, H-2′,6′ Ar), 8.22 (d, *J*= 7.2 Hz, 1H, H-6 Ar), 11.31 (s, 1H, NH, exchanged with D_2O), 11.53 (s, 1H, NH, exchanged with D_2O). Anal. Calcd. for $C_{16}H_{15}N_5O$ (293.32): C, 65.52; H, 5.15; N, 23.88. Found: C, 65.59; H, 5.12; N, 23.98.

S4.13. General procedure for synthesis of 4-(un)substituted phenyl-2,5,10,11-tetrahydropyrido[2,3-g]pyrazino[4,3-e]1,2,4-triazin-11-ones (series H, 12a-d):

Bromine (1.60 g, 0.53 ml, 0.01 mol) in glacial acetic acid (0.6 ml) was added dropwise to solution of compound **9a-d** (0.01 mol) in glacial acetic acid (9 ml) containing sodium acetate (2.46 g, 0.03 mol) at room temperature. The mixture was stirred for 3 h at room temperature then poured onto cold water (50 ml). The formed precipitate was filtered, washed with water (10 ml) and dried. The crude product was crystallized from acetic acid.

S4.13.1. 4-Phenyl-2,5,10,11-tetrahydropyrido[2,3-g]pyrazino[4,3-e]1,2,4-triazin-11-one (12a):

Yield 80%, mp 219-221°C, IR (KBr) [ʋ/cm^{-1}]: 3365, 3355 (2NH), 3035 (CH Ar), 1760 (C=O), 1608, 1558, 1541, 1521, 1508 (C=N, NH, C=C). ^{1}HNMR (400 MHz, DMSO-d_6): δ= 6.01 (s, 1H, H-1 Ar), 6.99 (t, 1H, H-8 Ar), 7.01 (d, *J*= 6.4 Hz, 1H, H-9 Ar), 7.29-7.41 (m, 3H, H-3′,4′,5′ Ar), 7.63 (d, *J*= 7.2 Hz, 2H, H-2′,6′ Ar), 7.91 (d, *J*= 6.0 Hz, 1H, H-7 Ar), 11.29 (s, 1H, NH, , exchanged with D_2O), 11.52 (s, 1H, exchanged with D_2O). Anal. Calcd. for $C_{15}H_{11}N_5O$ (277.28): C, 64.97; H, 4.00; N, 25.26. Found: C, 65.13; H, 4.06; N, 25.49.

S4.13.2. 4-(4-Chlorophenyl)-2,5,10,11-tetrahydropyrido[2,3-g]pyrazino[4,3-e]1,2,4-triazin-11-one (12b):

Yield 75%, mp 222-225 °C, IR (KBr) [ʋ/cm^{-1}]: 3390, 3385 (2NH), 3032 (CH Ar), 1680 (C=O), 1635, 1608, 1593, 1570, 1558 (C=N, NH, C=C). ^{1}HNMR (400 MHz, DMSO-d_6): δ= 5.99 (s, 1H, H-1 Ar), 6.86 (t, 1H, H-8 Ar), 7.02 (d, *J*= 8.1 Hz, 1H, H-9 Ar), 7.33 (d, *J*= 7.0 Hz, 2H, H-3′,5′ Ar), 7.62 (d, *J*= 6.5 Hz, 2H, H-2′,6′ Ar), 8.22 (d, *J*= 6.3 Hz, 1H, H-7 Ar), 11.31 (s, 1H, NH, exchanged with D_2O), 11.53 (s, 1H, NH, exchanged with D_2O). Anal. Calcd. for $C_{15}H_{10}ClN_5O$ (311.73): C, 57.79; H, 3.23; N, 22.47. Found: C, 57.96; H, 3.19;N, 22.63.

S4.13.3. 4-(4-Nitrophenyl)-2,5,10,11-tetrahydropyrido[2,3-g]pyrazino[4,3-e]1,2,4-triazin-11-one (12c):

Yield 86%, mp 222-224°C, IR (KBr) [ʋ/cm^{-1}]: 3410, 3393 (2NH), 3041 (CH Ar), 1674 (C=O), 1654, 1635, 1600, 1568, 1558 (C=N, NH, C=C), 1521, 1346 (NO_2). ^{1}HNMR (400 MHz, DMSO-d_6): δ= 6.21 (s, 1H, H-1 Ar), 6.62 (t, 1H, H-8 Ar), 6.84 (d, *J*= 7.2 Hz, 1H, H-9 Ar), 7.56 (d, *J*= 8.1 Hz, 1H, H-7 Ar), 7.91-8.10 (m, 4H, H-2′, 3′, 5′, 6′ Ar), 11.33 (s, 1H, NH, exchanged with D_2O), 11.50 (s, 1H, NH, exchanged with D_2O). Anal. Calcd. for $C_{15}H_{10}N_6O_3$ (322.28): C, 55.90; H, 3.13;N, 26.08. Found: C, 56.03; H, 3.17; N, 26.32.

S4.13.4. 4-(4-Methoxyphenyl)-2,5,10,11-tetrahydropyrido[2,3-g]pyrazino[4,3-e]1,2,4-triazin-11-one (12d):

Yield 66%, mp 229-231°C, IR (KBr) [ʋ/cm^{-1}]: 3415, 3388 (2NH), 3020 (CH Ar), 2933, 2841 (CH Aliph), 1674 (C=O), 1633, 1597, 1575, 1558, 1508 (C=N, NH, C=C). ^{1}HNMR *(300 MHz, DMSO-d_6): δ= 3.72 (s, 3H, OCH_3), 5.82 (s, 1H, H-1 Ar), 6.54 (t, 1H, H-8 Ar), 6.96 (d, *J*= 6.0 Hz, 1H, H-9 Ar), 7.03-7.40 (m, 4H, H-2′, 3′, 5′, 6′ Ar), 7.67 (d, *J*= 7.3 Hz, 1H, H-7 Ar), 11.33 (s, 1H, NH, exchanged with D_2O), 11.51 (s, 1H, NH, exchanged with D_2O). Anal. Calcd. for $C_{16}H_{13}N_5O_2$ (307.31): C, 62.53; H, 4.26; N, 22.79. Found: C, 62.64; H, 4.32; N, 22.94.

S5. Molecular docking

S5.1. Preparing the PDE5 protein structure for molecular docking

The PDB file (PDB ID: 3HC8) [2] was visualized by MOE software. The hydrogen atoms were placed and the overall lowest potential configuration energy was determined and identified. Both ligand and the active site pocket were isolated and visualized through molecular surface tool.

S5.2. Validation of docking protocol of reference ligand with the active site

The reference ligand (**t48**) was considered in the validation of docking process with the active site of phosphodiesterase enzyme. The Rigid Protein Docking protocol was used in the validation step. Triangle Matcher method (bond rotation method) was used in order to generate the reference ligand conformations. The produced conformations were ranked with the London dG scoring function. The minimization of conformations energies was accomplished with Forcefield functional form. The identified conformations were rescored with GBVI/WSA dG scoring function (S, kcal/mol).

S5.3. Docking of test set

The test set (series **A–H**) databases were selected as ligand atoms in the molecular docking procedure using the same setting for the reference ligand (**t48**, PDB ID: PD4).

S5.4. Analyzing the docking results

The best conformers for test set (lowest binding score and RMSD values) were selected to be visualized in the PDE5 active site pocket. The 2D and 3D interactions of each conformer were pictured in order to identify the binding modes with the possible interactions.

S6. Molecular dynamic (MD) simulation

Protein-ligand complex MD simulation was conducted to validate the molecular docking results. The binding complex of the most potent derivatives among the tested compounds (**11b**) was selected for molecular dynamic analysis. The crystal structure of the human PDE5 in complex with **t48** (PDB ID: 3HC8 [2]) was used as a control. The entire co-crystallized protein-ligand complex was used as a positive control of a true active binding mode, whereas the unbound protein without co-crystallized ligand – negative control. A total of six MD simulations, 50 ns each, were conducted. GROningen MAchine for Chemical Simulations (GROMACS 2016.4) was used to carry out the MD simulations [12]. The MD systems for each simulation were set up using the CHARMM22 forcefield [13-15] and the SwissParam web service was used to generate the ligand topology and parameters compatible with CHARMM and GROMACS [16]. The full system consisted of a dodecahedral box, solvated with TIP3P water molecules at a 10 Å edge distance and neutralized with Na^+ and Cl^- ions.

To remove any steric clashes, energy minimization was carried out using the steepest descent minimization algorithm with a maximum of 50,000 steps and a target F_{max} of no greater than 100 kJ mol^{-1} nm^{-1}. These energy minimized systems were equilibrated in two steps: thermalization (in the NVT ensemble) at 300 K and pressurization (in the NPT ensemble) at 1 bar each for 50 ns. Throughout both equilibrations, only the solvent molecules were permitted to freely move to ensure proper equilibration in the system while all other atoms were restrained. The particle mesh Eshwald method with a 12 Å cut-off and 16 Å Fourier spacing method were used to obtain the long-range electrostatics. The six equilibrated systems (one unbound protein and five protein-ligand complexes) were subjected to 50 ns unrestrained production runs. The output trajectories were re-centered and analyzed using built-in GROMACS commands and the VMD software (University of Illinois at Urbana-Champaign, Urbana, IL, USA) [17].

S7. References

[1] J. Fiorito, F. Saeed, H. Zhang, A. Staniszewski, Y. Feng, Y.I. Francis, S. Rao, D.M. Thakkar, S.-X. Deng, D.W. Landry, Synthesis of quinoline derivatives: discovery of a potent and selective phosphodiesterase 5 inhibitor for the treatment of Alzheimer's disease, Eur J Med Chem, 60 (2013) 285-294.
[2] D.R. Owen, J.K. Walker, E.J. Jacobsen, J.N. Freskos, R.O. Hughes, D.L. Brown, A.S. Bell, D.G. Brown, C. Phillips, B.V. Mischke, Identification, synthesis and SAR of amino substituted pyrido [3, 2b] pyrazinones as potent and selective PDE5 inhibitors, Bioorg Med Chem Lett, 19 (2009) 4088-4091.
[3] D.N. Patel, L. Li, C.-L. Kee, X. Ge, M.-Y. Low, H.-L. Koh, Screening of synthetic PDE5 inhibitors and their analogues as adulterants: analytical techniques and challenges, J Pharmaceut Biomed, 87 (2014) 176-190.
[4] H. Zheng, Y. Wu, B. Sun, C. Cheng, Y. Qiao, Y. Jiang, S. Zhao, Z. Xie, J. Tan, H. Lou, Discovery of furyl/thienyl β-carboline derivatives as potent and selective PDE5 inhibitors with excellent vasorelaxant effect, Eur J Med Chem, 158 (2018) 767-780.
[5] X. Ge, M.-Y. Low, P. Zou, L. Lin, S.O.S. Yin, B.C. Bloodworth, H.-L. Koh, Structural elucidation of a PDE5 inhibitor detected as an adulterant in a health supplement, J Pharmaceut Biomed, 48 (2008) 1070-1075.
[6] E. Duarte-Silva, A.J.M. Chaves Filho, T. Barichello, J. Quevedo, D. Macedo, C. Peixoto, Phosphodiesterase-5 inhibitors: shedding new light on the darkness of depression?, Journal of Affective Disorders, (2019).
[7] M. Bollenbach, C. Lugnier, M. Kremer, E. Salvat, S. Megat, F. Bihel, J.-J. Bourguignon, M. Barrot, M. Schmitt, Design and synthesis of 3-aminophthalazine derivatives and structural analogues as PDE5 inhibitors: anti-allodynic effect against neuropathic pain in a mouse model, Eur J Med Chem, 177 (2019) 269-290.
[8] H.A. Flores Toque, F.B. Priviero, C.E. Teixeira, E. Perissutti, F. Fiorino, B. Severino, F. Frecentese, R. Lorenzetti, J.S. Baracat, V. Santagada, Synthesis and pharmacological evaluations of sildenafil analogues for treatment of erectile dysfunction, J Med Chem, 51 (2008) 2807-2815.
[9] R.O. Hughes, J.K. Walker, J.W. Cubbage, Y.M. Fobian, D.J. Rogier, S.E. Heasley, R.M. Blevis-Bal, A.G. Benson, D.R. Owen, E.J. Jacobsen, Investigation of aminopyridiopyrazinones as PDE5 inhibitors: Evaluation of modifications to the central ring system, Bioorg Med Chem Lett, 19 (2009) 4092-4096.
[10] R.O. Hughes, J.K. Walker, D.J. Rogier, S.E. Heasley, R.M. Blevis-Bal, A.G. Benson, E.J. Jacobsen, J.W. Cubbage, Y.M. Fobian, D.R. Owen, Optimization of the aminopyridopyrazinones class of PDE5 inhibitors: Discovery of 3-[(trans-4-hydroxycyclohexyl) amino]-7-(6-methoxypyridin-3-yl)-1-(2-propoxyethyl) pyrido [3, 4-b] pyrazin-2 (1H)-one, Bioorg Med Chem Lett, 19 (2009) 5209-5213.
[11] A.H. Abadi, B.D. Gary, H.N. Tinsley, G.A. Piazza, M. Abdel-Halim, Synthesis, molecular modeling and biological evaluation of novel tadalafil analogues as phosphodiesterase 5 and colon tumor cell growth inhibitors, new stereochemical perspective, Eur J Med Chem, 45 (2010) 1278-1286.
[12] Abraham, Mark James, Teemu Murtola, Roland Schulz, Szilárd Páll, Jeremy C. Smith, Berk Hess, and Erik Lindahl. "GROMACS: High performance molecular simulations through multi-level parallelism from laptops to supercomput-ers." SoftwareX 1 (2015): 19-25.
[13] MacKerell Jr, Alex D., Donald Bashford, M. L. D. R. Bellott, Roland Leslie Dunbrack Jr, Jeffrey D. Evanseck, Martin J. Field, Stefan Fischer et al, All-atom empirical potential for molecular modeling and dynamics studies of proteins, J. Phys. Chem., 1998, 102, 3586-3616.
[14] MacKerell Jr, Alexander D., Michael Feig, and Charles L. Brooks, Improved treatment of the protein backbone in em-pirical force fields, J. Am. Chem. Soc., 2004, 126, 698-699.
[15] Mackerell Jr, Alexander D., Michael Feig, and Charles L. Brooks, Extending the treatment of backbone energetics in protein force fields: Limitations of gas-phase quantum mechanics in reproducing protein conformational distributions in molecular dynamics simulations, J. Comput. Chem., 2004, 25, 1400-1415.

[16] Zoete, Vincent, Michel A. Cuendet, Aurélien Grosdidier, and Olivier Michielin, SwissParam: a fast force field genera-tion tool for small organic molecules, J. Comput. Chem., 2011, 32, 2359-2368.
[17] Humphrey, William, Andrew Dalke, and Klaus Schulten, VMD: visual molecular dynamics, J. Mol. graphics, 1996, 14, 33-38.
